# *De Novo* Assembly of a High-Quality Reference Genome for the Horned Lark (*Eremophila alpestris*)

**DOI:** 10.1534/g3.119.400846

**Published:** 2019-12-18

**Authors:** Nicholas A. Mason, Paulo Pulgarin, Carlos Daniel Cadena, Irby J. Lovette

**Affiliations:** * Fuller Evolutionary Biology Program, Cornell Lab of Ornithology,; † Department of Ecology and Evolutionary Biology, Cornell University, Ithaca, New York 14853,; ‡ Museum of Vertebrate Zoology, University of California, Berkeley, California, 94720,; § Laboratorio de Biología Evolutiva de Vertebrados, Departamento de Ciencias Biológicas, Universidad de Los Andes, Bogotá, Colombia, and; ** Facultad de Ciencias y Biotecnología, Universidad CES, Medellín, Colombia

**Keywords:** Alaudidae, ALLPATHS-LG, *Eremophila alpestris*, genome assembly, horned lark

## Abstract

The Horned Lark (*Eremophila alpestris*) is a small songbird that exhibits remarkable geographic variation in appearance and habitat across an expansive distribution. While *E. alpestris* has been the focus of many ecological and evolutionary studies, we still lack a highly contiguous genome assembly for the Horned Lark and related taxa (Alaudidae). Here, we present CLO_EAlp_1.0, a highly contiguous assembly for *E. alpestris* generated from a blood sample of a wild, male bird captured in the Altiplano Cundiboyacense of Colombia. By combining short-insert and mate-pair libraries with the ALLPATHS-LG genome assembly pipeline, we generated a 1.04 Gb assembly comprised of 2713 scaffolds, with a largest scaffold size of 31.81 Mb, a scaffold N50 of 9.42 Mb, and a scaffold L50 of 30. These scaffolds were assembled from 23685 contigs, with a largest contig size of 1.69 Mb, a contig N50 of 193.81 kb, and a contig L50 of 1429. Our assembly pipeline also produced a single mitochondrial DNA contig of 14.00 kb. After polishing the genome, we identified 94.5% of single-copy gene orthologs from an Aves data set and 97.7% of single-copy gene orthologs from a vertebrata data set, which further demonstrates the high quality of our assembly. We anticipate that this genomic resource will be useful to the broader ornithological community and those interested in studying the evolutionary history and ecological interactions of larks, which comprise a widespread, yet understudied lineage of songbirds.

The Horned Lark (*Eremophila alpestris*) is a widespread species of songbird that occupies grasslands, tundras, deserts, and other sparsely vegetated habitats on five continents (Beason 1995). As is characteristic of most species in the family Alaudidae, *E. alpestris* is a terrestrial species that nests on the ground and relies on camouflage to avoid predation by avian predators (Donald *et al.* 2017). The Horned Lark has been studied extensively in terms of geographic variation and systematics (Behle 1942; Johnson 1972), population genetics (Drovetski *et al.* 2006, 2014; Mason *et al.* 2014; Ghorbani *et al.* 2019), physiological adaptations (Trost 1972), breeding biology (de Zwaan *et al.* 2019), and responses to human activity, such as agriculture (Mason and Unitt 2018) and wind energy (Erickson *et al.* 2014), among other focal areas. Despite extensive past and ongoing research involving *E. alpestris* and other alaudids, we lack a highly contiguous reference genome for the species and the family as a whole (but see Dierickx *et al.* 2019). Generating genomic resources for the Horned Lark and related taxa will enable studies linking phenotypic and genetic variation (Kratochwil and Meyer 2015; Hoban *et al.* 2016), chromosomal rearrangements (Wellenreuther and Bernatchez 2018), and many other avenues of future genomic research for non-model organisms (Ellegren 2014).

Here, we describe CLO_EAlp_1.0, a new genomic assembly that we built with DNA extracted from a wild, male lark captured from a demographically small and geographically isolated population near Toca, Boyacá, Colombia. We sampled this individual and population because it had high *a priori* likelihood of high homozygosity compared to larks elsewhere with much larger effective population sizes and variable patterns of connectivity to adjacent populations. To generate this *de novo* assembly, we used the ALLPATHS-LG pipeline (Butler *et al.* 2008; Gnerre *et al.* 2011). Given the lack of genomic resources currently available for Alaudidae, we hope this *de novo* assembly will inspire and facilitate future studies on the genomic biology of larks—a widespread, diverse lineage of songbirds.

## Materials and Methods

### Sample collection, DNA extraction, and sequencing

We captured a male *E. alpestris* (EALPPER07; NCBI BioSample SAMN12913182) approximately 170 km NE of Bogotá, Colombia near the town of Toca on the shores of the Embalse de La Copa in the Altiplano Cundiboyacense of the Boyacá department (5.623299, -73.184156). This population is small and represents a subspecies (*E. a. peregrina*) that is geographically isolated from other populations of larks, the nearest population of which is in Oaxaca, Mexico. The Colombian subspecies of Horned Lark likely underwent a population bottleneck upon colonizing the distant, high-elevation plateaus of the Altiplano Cundiboyacense region and therefore probably has high homozygosity compared to other populations, which is preferable for *de novo* genome assembly. We collected blood from the brachial vein, from which we subsequently extracted genomic DNA with a Gentra Puregene Blood Kit (Qiagen, Hilden, Germany) following the manufacturer’s protocol. We confirmed the sex of the individual using PCR amplification (Chu *et al.* 2015). After running the sample on a 1% agarose gel to confirm the presence of high molecular weight DNA, we sent the extraction to the Cornell Weil Medical School (New York, USA), where they generated a 180 bp fragment library, a 3 kb mate-pair library and a 8 kb mate-pair library. We sequenced the 180 bp library across two lanes and combined the 3 kb and 8 kb mate-pair libraries on another lane of Illumina HiSeq 2500 to perform 100 bp paired-end sequencing.

### Genome assembly, polishing, and assessment

We assembled the genome with ALLPATHS-LG v52415 (Butler *et al.* 2008; Gnerre *et al.* 2011). We did not perform additional adapter removal or quality filtering with the short-insert 180 bp libraries because ALLPATHS-LG has built-in steps that remove low-quality and adapter-contaminated reads (Butler *et al.* 2008). Once the initial assembly had finished, we aligned the short-insert and mate-pair libraries back to the assembly genome using bwa 0.7.17-r1188 (Li and Durbin 2009) and samtools v1.9 (Li *et al.* 2009) and then performed three iterations of scaffold polishing using pilon v1.22 (Walker *et al.* 2014) with default parameters. Once scaffold polishing had finished, we ordered and correspondingly renamed the scaffolds with respect to decreasing scaffold size using SeqKit v0.7.2 (Shen *et al.* 2016). We assessed the contiguity of the *de novo* genome using the function stats.sh from BBMap v38.73 (Bushnell 2014) and estimated genome completeness with BUSCO v3 (Simão *et al.* 2015; Waterhouse *et al.* 2018) alongside HMMER v3.1b2 (Finn *et al.* 2011) and BLAST+ v2.7.1 (Camacho *et al.* 2009) to identify single-copy orthologous gene sets among birds and vertebrates. We subsequently submitted our genome to the NCBI genome submission portal, which performs an additional scan for contaminants, including adapter contamination, and removed any additional contaminant sequences that were detected.

### Mitochondrial genome assembly

We also assembled the mitochondrial genome for the same individual (EALPPER07) with NOVOplasty v3.7 (Dierckxsens *et al.* 2017) using a ND2 sequence (GenBank Accession KF743558) from a previous study (Mason *et al.* 2014) as the initial seed to begin the assembly process.

### Data availability

Raw output from sequencing runs and the final assembly, CLO_EAlp_1.0, are available from NCBI (BioProject PRJNA575884). Short-fragment and mate-pair libraries are also available from the NCBI SRA (SUB6392689). Outputs from BUSCO and BBMap analyses are available from figshare (https://doi:10.6084/m9.figshare.9956063; https://doi:10.6084/m9.figshare.9956042). Supplemental material available at figshare: https://doi.org/10.25387/g3.9992360.

## Results and Discussion

Taken together, the three lanes of Illumina HiSeq 2500 sequencing generated 1.59 × 10^9^ total reads (∼134x estimated coverage of a 1.2 Gb genome), including 5.45 × 10^8^ paired-end reads for the 180 bp short-insert libraries, 1.24 × 10^8^ paired-end reads for the 3 kb mate-pair library, and 1.27 × 10^8^ paired-end reads for the 8 kb mate-pair library. Following scaffold polishing, the finalized CLO_EAlp_1.0 assembly consisted of 2713 scaffolds that totaled 1.04 Gb. The largest scaffold was 31.81 Mb while the scaffold N50 was 9.42 Mb and scaffold L50 was 30 (Table 1). The assembly consisted of 23,684 contigs, including a largest contig size of 1.69 Mb, a contig N50 of 193.81 kb, and a contig L50 of 1429. The average GC content of the assembly was 42.23%, which is similar to other birds (Jarvis *et al.* 2014; Botero-Castro *et al.* 2017), while the *de novo* genome assembly included 94.5% of single-copy orthologs from the Aves data set and 97.7% of the Vertebrata data set as identified by BUSCO (Table 2). The mitochondrial assembly pipeline generated a single mtDNA contig of 14 kb.

**Table 1:** *De novo* genome assembly metrics estimated using BBMap

Assembly Statistic	CLO_EAlp_1.0
# scaffolds / contigs	2713 / 23684
Largest scaffold / contig	31.81 Mb / 1.69 Mb
Total length	1.04 Gb
Scaffold / contig N50	9.42 Mb / 193.81 kb
Scaffold / contig N90	1.20 Mb / 31.02 kb
Scaffold / contig L50	30 / 1429
Scaffold / contig L90	141 / 6205
# N’s per 100 kbp	3472.35
GC (%)	42.23

**Table 2:** Output from BUSCO analyses to assess genome completeness by searching for single-copy orthologs from aves and vertebrata datasets

	Aves	Vertebrata
Complete BUSCOs	4645 (94.5%)	2530 (97.7%)
Complete and single-copy BUSCOs	4590 (93.4%)	2518 (97.4%)
Complete and duplicated BUSCOs	55 (1.1%)	12 (0.5%)
Fragmented BUSCOs	162 (3.3%)	36 (1.4%)
Missing BUSCOs	108 (2.2%)	20 (0.7%)
Total BUSCO groups searched	4915	2586

We opted not to assemble pseudochromosomes by aligning our *de novo* genome to an existing chromosome-level genome assembly (*e.g.*, Zebra Finch (*Taeniopygia guttata*). While birds generally exhibit strong synteny (Derjusheva *et al.* 2004), avian sex chromosomes and microchromosomes are often comprised of extensive rearrangements (Volker *et al.* 2010). Our assembly could be further improved such that scaffolds match full chromosomes through strategies such as Hi-C (Burton *et al.* 2013) or ultra-long read sequencing technology (Ma *et al.* 2018). Functional annotation of our assembly could also be improved by generating RNA-Seq and protein libraries specifically for larks. Nonetheless, CLO_EAlp_1.0 represents a large step forward toward leveraging the natural history of larks and advanced sequencing technology to further understand avian biology.
